# ALONE TOGETHER: SOCIAL ISOLATION AND LONELINESS ACROSS COVID-19

**DOI:** 10.1093/geroni/igad104.0264

**Published:** 2023-12-21

**Authors:** Emorie Beck, Anthony Ong, Eileen Graham

**Affiliations:** University of California, Davis, Davis, California, United States; Cornell University, Ithaca, New York, United States; Northwestern University, Chicago, Illinois, United States

## Abstract

The COVID-19 pandemic has resulted in a worldwide increase in the number of adults who are socially isolated and experiencing high levels of loneliness. Although research has focused on social isolation (the lack of social contact) and loneliness (the perception of being alone), few longitudinal studies have examined isolation and loneliness together in the context of the COVID-19 pandemic. We introduce a novel index of resilience to loneliness (social asymmetry), which captures the degree of congruence or discordance between subjective feelings of loneliness and objective social contact (social isolation). Using seven longitudinal samples (Total N > 75,000) across three continents, we examined trajectories of social asymmetry across the onset of the pandemic using Bayesian multilevel discontinuous growth models. These allowed us to demonstrate differences in level and change of social asymmetry across the onset of the pandemic. Levels and change tended toward resilience over time but did not show significant changes across the onset of the pandemic. However, these results were not consistent across samples, indicating heterogeneity across countries, age groups, and more. We discuss how examining COVID-related changes in social asymmetry advances our understanding of (1) the extent to which broad societal challenges can induce short- and long-term impacts on trajectories of resilience and (2) what factors account for heterogeneity in adaptive responses to adversity.

